# Mechanistic links between TMEM106B and TDP‐43 dysfunction in AD‐related dementias

**DOI:** 10.1002/alz.090873

**Published:** 2025-01-03

**Authors:** Mercedes Prudencio

**Affiliations:** ^1^ Mayo Clinic, Jacksonville, FL USA

## Abstract

**Background:**

Inclusions of TAR DNA binding protein of 43kDa (TDP‐43) constitute the main characteristic pathology in the majority (∼97%) of amyotrophic lateral sclerosis (ALS) cases and approximately 50% of patients with frontotemporal lobar degeneration (FTLD). TDP‐43 is a nuclear RNA binding protein; however, in disease, it becomes hyperphosphorylated and/or insoluble, hindering its nuclear function in maintaining RNA homeostasis. Importantly, the incidence of TDP‐43 proteinopathy extends to aging brains (LATE) and may be concomitant with Alzheimer’s disease (AD) neuropathological changes (LATE/AD) in up to 70% of AD patients. Interestingly, LATE/AD cases are characterized by worse memory and greater hippocampal atrophy than AD alone. Therefore, TDP‐43 dysfunction could have diverse, disease‐specific effects on pathogenesis, making it imperative to elucidate the mechanism of TDP‐43 deposition in the brain and its role in AD‐related dementias (ADRDs).

**Method:**

We used standardized molecular biology techniques and single‐nuclei RNA sequencing to evaluate TDP‐43 dysfunction across brain regions in both LATE/AD and FTLD‐TDP, identify common and distinct molecular mechanisms involved in each ADRD, as well as assess the relevance of *TMEM106B* rs3173615, a genetic variation linked to risk of FTLD haplotype, in TDP‐43 pathology.

**Result:**

We detected misspliced, TDP‐43‐regulated cryptic RNAs in the amygdala and hippocampus of LATE/AD and FTLD‐TDP. FTLD‐TDP, but not LATE/AD, frontal cortex also showed significant accumulation of these aberrant transcripts. The topographic distribution of cryptic RNAs mimicked that of insoluble, phosphorylated TDP‐43, regardless of TDP‐43 subtype classification. Despite common misregulated transcripts in these ADRDs, we also identified unique transcriptome changes, even within each TDP‐43 subtype. Finally, we observed that TMEM106B core deposition, which is linked to the presence of the risk *TMEM106B* rs3173615 haplotype, was associated with enhanced TDP‐43 dysfunction and pathology, and interactome data suggested a role for TMEM106B core filaments in impaired RNA transport, local translation, and endolysosomal function in FTLD‐TDP.

**Conclusion:**

Our results emphasize the use of cryptic RNAs to identify cases with TDP‐43 pathology, and raises the possibility that unique transcriptome signatures may further distinguish FTLD‐TDP from LATE/AD and within different TDP‐43 subtypes. We showed a relationship between increased TMEM106B core deposition and greater susceptibility to TDP‐43 dysfunction, potentially through dysregulation of endolysosomal function.

